# Lip Height Improvement during the First Year of Unilateral Complete Cleft Lip Repair Using Cutting Extended Mohler Technique

**DOI:** 10.1155/2012/206481

**Published:** 2012-12-20

**Authors:** Cassio Eduardo Raposo-Amaral, André Pecci Giancolli, Rafael Denadai, Frederico Figueiredo Marques, Renato Salazar Somensi, Cesar Augusto Raposo-Amaral, Nivaldo Alonso

**Affiliations:** ^1^Institute of Plastic and Craniofacial Surgery, SOBRAPAR, Campinas, SP, Brazil; ^2^Plastic Surgery Division, Department of Surgery, Universidade de São Paulo (USP), São Paulo, SP, Brazil

## Abstract

*Objective*. To compare the cutaneous lip height at early and late postoperative periods and to objectively determine the average amount of lip height improvement during the first year of unilateral complete cleft lip repair using Cutting extended Mohler technique. *Methods*. In this prospective cohort study, 26 unilateral complete cleft patients and 50 noncleft subjects were included. Photographs were taken between 12 and 16 weeks (T1) and also taken between 12 and 13 months after surgery (T2). The cutaneous lip height distance (photogrammetric lip analysis) obtained in these two periods of time were measured and statistically analyzed. *Results*. The average lip heights were 24% ± 9% in T1 and 8% ± 6% in T2 (*P* < 0.01). The average lip height asymmetry in the noncleft individuals was 4.52% ± 1.89%. *Conclusion*. Since all principles to obtain a symmetrical Cupid's bow were performed, the postoperative pull-up of Cupid's bow is probably owed to the scar contracture, which improves by 2 times during the first year after surgery.

## 1. Introduction

 Ralph Millard revolutionized the treatment of cleft lip by describing the innovative principles to repair a unilateral cleft lip, that allows surgeons around the globe to treat patients with different racial characteristics [1–4]. Consequently, his principles remain as a foundation to the development of surgical strategies and tactics to improve the results in the cleft lip repair worldwide [5–7]. Mohler [8] used Millard's principles to develop his own technique, that adds a more vertical incision in the cleft philtrum column, creating a final faint scar that represent a mirror image of the contralateral philtrum column. In the Mohler technique [8], the Millard's C-flap is used to fill the gap created by the downward rotation of the cleft lip segment, instead of the lateral advancement segment as proposed by Millard [5]. Thus, a short lip height may be produced as a consequence of these maneuvers (straight-line scar and absence of the lateral advancement segment fulfilling the medial gap after the back-cut incision). 

Cutting's modifications of the Mohler technique are the following: (1) an extension of the medial incision toward the columella, (2) the Millard's back-cut incision never passes the noncleft philtral column, (3) a more vertical incision than that described by Millard, that creates a, and (4) wider C-flap that fills the medial rotation defect [9]. Cutting and Dayan [9] subsequently analyzed cleft patients who underwent a cheiloplasty, using the Cutting extend Mohler technique, in order to respond whether this technique produces a short lip height and lip width in two different postoperative periods of time. Cleft and noncleft distances in each patient were measured preoperatively and postoperatively at two different periods of time (1 to 13 months and at 2 years or more) and statistically compared [9]. Their data did not show statistically significant changes in lip height over time and showed statistically increased cleft-side lip width over time [9].

Interestingly, some surgeons [10–13] have observed the lip height changes using Millard and other cheiloplasty techniques. Even Cutting and Dayan [9] acknowledged in the same study that the peak of Cupid's bow pulls up short at the sixth postoperative week in some patients who had undergone surgery. The authors [9] concluded that the short lip height usually normalizes at 12 months after the surgery. In a small percentage of their patients, a cleft lip height remained short, possibly owing to the scar contracture of the straight-line closure [9]. Even the authors [9], although shown that the lip height does not statistically change over time, recognized the importance of warning the parents regarding the phenomenon of scar contracture that pulls up Cupid's bow and shortens the lip around the sixth week in the postoperative period. 

We have been performing the Cutting extended Mohler technique and also, anecdotally, observed the decrease of the lip height in a latter period than Cutting and Dayan, that ranges from 12 to 16 weeks in the postoperative period. Interestingly, the majority of the parents also observed a short lip height at 12 to 16 weeks in the postoperative period in comparison to the period that comprehended the first week after surgery. Thus, we strongly believe that objective prospective data, based on the lip height measurement in this period of time (from 12 to 16 weeks after surgery) is necessary to counsel and calm the parents, giving them an average percentage of improvement during this time frame.

 Since Cutting and Dayan used a long time frame (from immediate postoperative period to 13 months postoperative period) in their study to analyze the lip height [9], we believe that a prospective study is necessary to objectively quantify the lip height in the period of time that ranges from 12 to 16 weeks, using a lower variation of time frame. Thus, we decided to restrict the time frame of the study performed by Cutting and Dayan [9] to verify if there is a statistical difference in the cutaneous lip height at early postoperative period (from 12 to 16 weeks), in comparison to a late postoperative period (between 12 and 13 months after surgery) in patients who underwent unilateral cleft lip repair, by modified Cutting extended Mohler technique without facial orthopedics. Additionally, this study aimed to objectively quantify the average amount of lip height improvement during the first year of surgery, which may ultimately be used to calm the parents during follow-up consultations and to determine the average amount of lip height discrepancy in a noncleft population. 

## 2. Patient and Methods

A prospective observational study was conducted of 35 nonsyndromic unilateral complete cleft lip patients only, who underwent primary cleft lip repair by modified Cutting extended Mohler technique [9], without facial orthopedics performed from 2008 to 2010. The inclusion criteria were all patients who presented unilateral complete cleft lip repair, who underwent surgery using a Cutting extended Mohler technique and who had more than a year of follow-up time. All patients, who did not return to follow-up consultation in this time frame (from 12 to 16 weeks) and at 12 to 13 months after surgery, were excluded from this study.

To determine whether perfect symmetry is encountered, data from 50 noncleft, Brazilian individuals were obtained. The children chosen to be controls were volunteers recruited from a group of 120 children with good general health and no visible facial asymmetry that had been previously selected from a local primary school by two plastic surgeons (not involved in the present study); 50 volunteers were allocated via a computer-generated process for the study group.

All subjects (cleft lip patients and healthy volunteers) were enrolled upon a consent form signed by their parents, in accordance with the Helsinki Declaration of 1975, as amended in 1983. A local institutional research ethics board approval was obtained for this study.

### 2.1. Photographic Documentation

Two-dimensional photographs were employed for the evaluation of the perioral region of the cleft patient's face. Photographic full face frontal view using the camera at the same level of the patient's head was standardized [14], prior to the study initiation by the first author and a professional photographer of the Institution. The photographs of cleft patients were taken at 12 to 16 weeks (T1), after surgery, and at 12 to 13 months after surgery (T2), while the photographs of noncleft individuals were performed in a single period. All photographs were taken at least 5 times in a professional studio with 3 flashes by the professional photographer. Only one photograph for each patient was chosen to execute the measurements.

The distance, (approximately 1 m) between the photographer and the patient, was marked with lines on the ground. All photographs were taken with a Nikon D200 digital camera and 100 mm Nikon macro lens in a 1 : 1 ratio (Nikon Corp, Tokyo, Japan). All photographs were archived for later analysis and codified using randomly assigned numbers by one of the investigators. 

### 2.2. Standardization of the Anatomic Landmarks

The anatomical landmarks used for measurements were defined preoperatively. Prior to the study, two surgeons (not involved in the operation) were taught the anatomic landmarks where the measurements should be done. The anatomical landmarks were defined and marked with a red dot by one of them, and the measurements were consecutively reproduced by both two weeks later.

### 2.3. Anatomic Landmarks

The cutaneous lip height was defined as the distance from each peak of Cupid's bow (transition between the white roll and vermillion) to the virtual plane generated by the initial lateral aspects of the collumelar base [15–17].

### 2.4. Photogrammetric Analysis

Photogrammetric lip analysis was performed from a frontal view processed by Photoshop CS4 extended (Adobe System, Inc., San Jose, CA, USA). 

To calculate the difference in the cutaneous lip height between the two sides of the cleft population, the noncleft lip height (*L*1) was compared with the cleft lip height (*L*2) in each patient, using the following formula: Lip height difference (LHD) = *L*1 − *L*2/*L*1 × 100. For the noncleft individuals: LHD = length  of  the  longest  side − length  of  the  shorter  side/length  of  the  longest  side × 100. This formula yields a percent difference, which was calculated for each patient. The same formula to evaluate nasal alar contour was initially proposed by Wong et al. [18] and subsequently modified by Fudalej et al. [15]. The lip height difference was defined as an index of asymmetry of the lip.

The amount of lip height difference improvement in each cleft patient was determined by the difference in LHD at T2 and LHD at T1. A LDH equal to zero indicated perfect symmetry and any deviation from that determined asymmetry of the lip.

**Figure 1:**
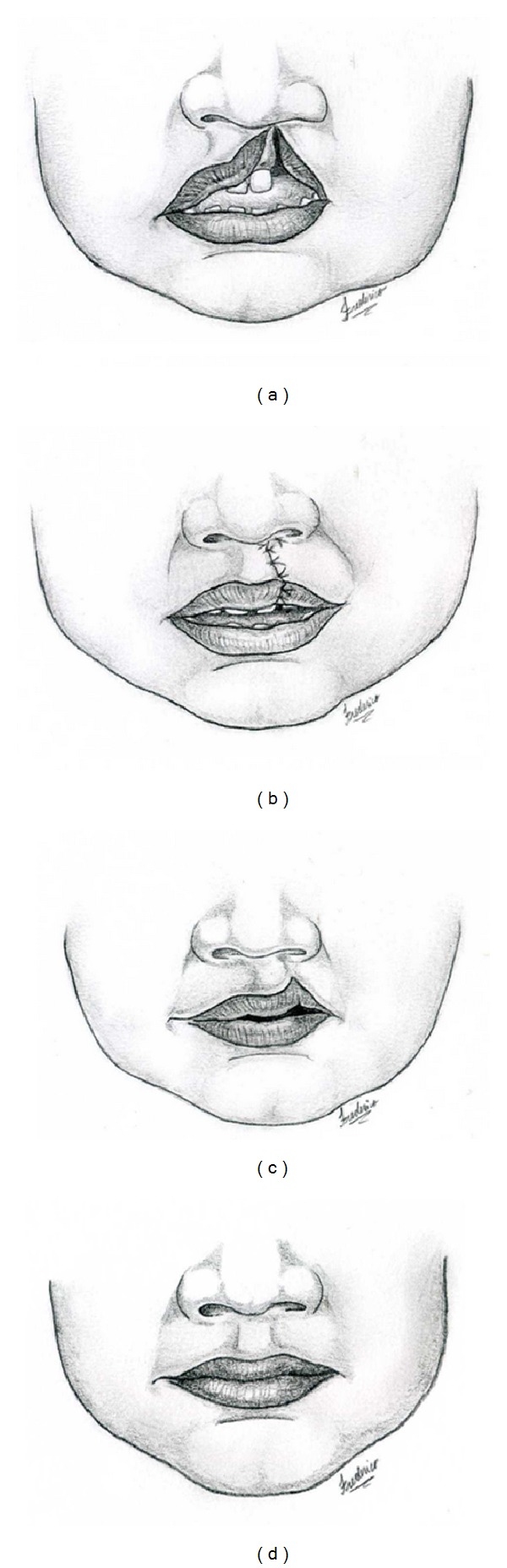
This drawing illustrates the sequence of preoperative 2-year-old cleft patient (a), the on-table result (b), the dynamic pull up of Cupid's bow at T1(c), and its average improvement at T2 (d). This study was designed to quantify the amount of lip movement and improvement during the first year after surgery.

The three asymmetry lip scores are descriptively presented using a four point scale: asymmetry ≤4%, from 5% to 10%, from 11 to 20%, and more than 20%. This lip score was adapted [19] to determine the lip asymmetry in the second period of time (T2) (Figure 1).

### 2.5. Statistical Analysis

In the descriptive analysis, the mean and standard deviations were used for metric variables, and percentages were given for categorical variables. All measurements related to upper lip height were summarized as means and standard deviations. Friedman tests were used to compare the measurements in the two postoperative periods of time. Person's correlation test was performed to correlate the measurements performed by the two observers. The Statistical Package for Social Sciences (SPSS version 16.0 for Windows, Chicago, IL, USA) was used for all statistical calculations. Values were considered significant for a confidence interval of 95% (*P* < 0.05).

## 3. Results

Twenty-six cleft patients (74.29%) and fifty noncleft patients were included in the study. Nine cleft patients (25.71%) were excluded for not presenting themselves to the craniofacial clinics at proper timing. The average age at the operation was 6.31 ± 8.64 months (range from 3 to 48 months). The noncleft individuals had neither syndromes nor midface hypoplasia affecting the soft tissue (lip) metrics. The average age of non-cleft individuals was 38.4 ± 13.07 months (range from 7 to 48 months).

**Figure 2:**
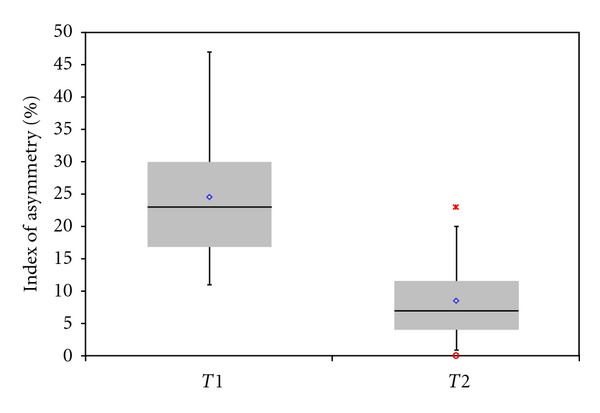
Boxplot showing the dispersion of the values of index of asymmetry based on objective evaluation. The index of asymmetry in period T1 (2 to 6 months after surgery) was higher (*P* < 0.01) than the index in period T2 (12 to 13 months after surgery). The diamond symbol represents the mean value. The heavy line is the median. The bars represent the data range. The symbols “*” and “°” indicate the outliers.

**Figure 3:**
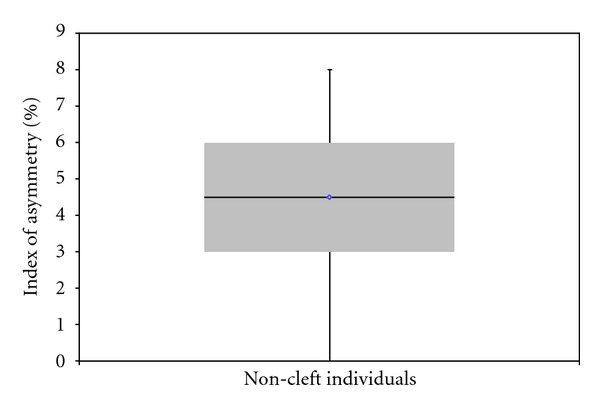
Boxplot showing the dispersion of the values of index of asymmetry based on objective evaluation. The diamond symbol represents the mean value. The heavy line is the median. The bars represent the data range.

**Figure 4:**
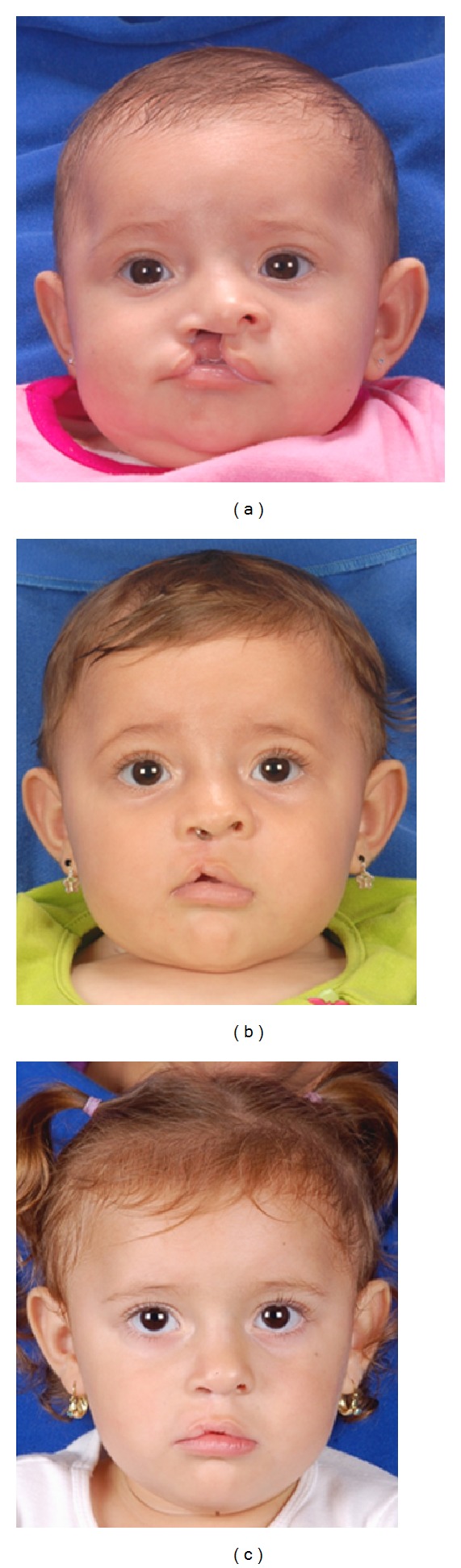
(a) A 3-month-old, complete cleft patient who underwent a cleft lip repair using the Cutting extended Mohler technique. (b) The initial result at T1 showing the pull up of Cupid's bow, owing to the scar contraction in this period of time. (c) The T2 result shows a better positioning of Cupid's bow and satisfactory lip height in the cleft side.

The average lip heights (index of asymmetry of the cutaneous lip height) in the cleft patients were 24% ± 9% in T1 and 8% ± 6% in T2. The comparison between the two postoperative periods of time showed statistically significant difference (*P* < 0.01). The average improvement of the lip height during the first year after surgery was 16%. Seven patients (26.92%) presented asymmetry of less or equal than 4%, 11 (42.31%) patients presented asymmetry from 5% to 10%, 6 (23.08%) patients from 11% to 20%, and 1 patient (3.85%) more than 20% (Figures 2, 3, and 4; Table 1).

The average lip height asymmetry in the noncleft individuals was 4.52% ± 1.89% (Table 2).

The reliability score of interobserver measurement was 0.9, indicating great similarity of the measurements. No additional complication was seen in the early and late postoperative periods. 

## 4. Discussion

Andewalla and Narayanan [11] also observed that a straight-line part of the Millard incision often contracts and pulls Cupid's bow up in the first few months and it descends at one year after surgery, without the need of a secondary surgery. Thus, we designed this study to objectively quantify the amount of pulls up of Cupid's bow, when it appeared to be maximum. The percentage yielded by the formula used in this study was interpreted as an index of asymmetry of the lip. Thus, our data showed that the average asymmetry of the lip at T1 was 24%, in comparison to a more favorable index of 8% at T2. The results of this study demonstrate statistically significant improvement in the cutaneous lip height over time, meaning that the symmetry of the lip improves by 2 times. This findings corroborate to our primary hypotheses that the lip pulls up short at the period of time that ranges from 12 to 16 months and subsequently improves. Considering that all principles of cleft lip repair were performed to obtain a symmetric Cupid's bow, this vertical decrease of the lip height is probably owed to scar contracture, that is apparently maximum when straight-line lip repair is advocated. We hypothesized that the lips that remained short at 1 year after surgery will descend in a longer follow-up period, since we respect the principles of the rotation advancement and the on-table result showed complete symmetry between the cleft and noncleft sides. 

The key element in these principles is the proper rotation of the cleft segment, achieved by the back-cut incision [20]. One should emphasize the release of the muscle and dermis instead of trimming the skin during this maneuver [20]. Another aspect to obtain a symmetric Cupid's bow is the preoperative markings on a fine-tip pen [21]. The Noordhoff's point has been used by surgeons worldwide to identify the optimal height of the lateral Cupid's bow and represent the greatest width of the vermilion border across from the white roll [22].

Before Millard's era, the simple straight-line closure introduced by Thompson [23], without using the principles of the rotation-advancement, were rejected by many surgeons [24, 25]. Some authors [26, 27] started closing the cleft lips using Z-plasts techniques that apparently would solve the problem of the scar contracture. On the other hand, at least one limb of the Z may obliterate the philtrum dimple, that is naturally concave. The natural concavity of the philtrum dimple can only be created without scars crossing this region. In the Cutting extended Mohler technique, the scar crosses only one set of the Langer's line at the collumelar base, further from the lowest and deepest region of the philtrum dimple [9]. The philtrum dimple can be created during the *orbicularis oris* muscle undermining and repair and final cutaneous closure [28]. An interposing orbicularis muscle repair proposed by Cutting and end-to-end muscle repair with vertical mattress sutures proposed by Mulliken helps evert the muscle to form the philtral ridge and accentuates the concavity of the philtum dimple [5]. An additional 1 mm deephitelization of the skin of the lateral segment during cutaneous closure may also contribute to its formation. The decrease of lip height discrepancy over the follow-up period is probably attributed to meticulous closure of the *orbicularis oris* muscle. Additionally, the upper medial portion of the *orbicularis oris* is fully trimmed and rotated downward to accentuate the proper rotation of the cleft segment and to help decrease the lip height discrepancy, improving lip symmetry in the follow-up period [5]. These maneuvers allow the surgeon to try a more straight-line incision that potentially simulates the position of the contralateral noncleft philtrum column [8]. The cutaneous closure may also play a role in the final lip height discrepancy. We have been using a nylon suture with an atraumatic needle to decrease the postoperative inflammation and scar contraction. We believe that the benefits of using them outweigh the disadvantages of stitch removal, usually accomplished under sedation in the operation room. 

 Even using all these principles, we observed an average of 8% lip asymmetry in one year follow-up period. However, measurements performed in 50 noncleft, Brazilian children using the same methodology determined the “normal” index of lip asymmetry. Interestingly, noncleft, Brazilian individuals may present 4% lip asymmetry, meaning that some degree of lip asymmetry is always expected, suggesting that one side of the face is rarely, completely identical to the contralateral side [29]. Interestingly, the Millard Society statement on its logo is “know the normal,” which may prove not to be the complete symmetry [30]. 

As also stated by Millard [31] in his treatise “*Cleft Craft*,” in the treatment of unilateral cleft lip, the normal side sets the standard and the ideal pattern to be simulated. All surgical effort has been made to have the length of the cleft philtrum column matched with length of the noncleft side. However, wide clefts with more than 10 mm of horizontal and vertical discrepancies in the alveolar region may turn the rotation maneuver somehow challenging to perform. 

Cutting and Dayan [9] did not show lip height discrepancy over the follow-up period and included in their cohort, patients with unilateral complete cleft previously treated with facial orthopedics. Although laboring, facial orthopedics has the advantage to approximate the palatal shelves and decrease the severity of the cleft defect, which may facilitate closure by decreasing its tension, that is ultimately related to scar contraction. Thus, further studies may identify the role of facial orthopedics on decreasing the tension of the final cutaneous suture, scar contraction, and lip height deficiency, especially in severe patients with wide clefts. Facial orthopedics, not performed in our patients, may be one variable that contributed to the remaining 8% of lip height discrepancy in our patients.

 In our study, we could quantify the exact amount of lip height in two different periods of time and demonstrate that the average asymmetry of the lip improves by 2 times during the first 12 months after surgery. Cutting and Dayan [9] also investigated the lip height at 2 years or more after surgery and found lower rate of lip asymmetry at this period of time. Since we evaluate the lip height at 12 to 13 months, it is possible that the lip continues to descend during the second year after the operation. Thus, further studies on this subject may elucidate this question.

 Our study may present some limitations. Three-dimensional distances performed by two-dimensional photography may carry an inherent bias [32], thus we exclusively measured the lip height distance and disregarded the lip width distance. The anatomic landmarks that allow the measurement of the lip height are almost at the same plane that exponentially decrease the inherent error of this methodology. The professional studio allowed a fast set of photographs and the possibility to adjust the proper positioning of the child's head and simultaneously have the pictures taken. Additionally, a lip height ratio was determined to avoid potential flaws generated by the parallax effect, caused by movement of the head and subtle modification of the distance between the patient and photographer. Digital, two-dimensional photographs still carry the advantages of being practical, cheap, and a noninvasive method [33]. However, proper equipment, a photography studio, and a professional photographer may help the standardization of the process and may decrease the inherent possibility of error during measurement [32]. Furthermore, as inherent errors from computer-based markings of two-dimensional photographs may exist [32], it is recommended that some time would be taken to become familiar with the software and the procedure for marking images, prior to data collection [33], as adopted in this study.

Although three-dimensional imaging indeed opened a new perspective in the facial measurements [32], it is limited by the current unavailability of equipment for routine clinical use, the training required and the cost of the equipment [33]. It is especially useful when the distance of two anatomical points located in two different planes is performed (e.g., tip of the nose and peak of Cupid's bow). The anthropometry of a child's face, performed by a caliper, lacks accuracy due to the usual lack of collaboration from children and eventually from parents, and it is highly operator-dependent.

Van Loon et al. [29] compared the measurements of the lip in complete unilateral cleft lip after primary cheiloseptoplasty to a noncleft control using 3D stereophotogrammetric analysis and stressed the difficulty to obtain near normal symmetrical relations. They [29] also emphasized, as our data showed in this study, that noncleft individuals also show some degree of asymmetry. Thus, all surgical efforts should be made to achieve the normal, which is not the perfect symmetry. 

## 5. Conclusion 

 Our data demonstrated lip height improvement during the first year of unilateral complete cleft lip repair using Cutting extended Mohler technique. Since all principles to obtain a symmetrical Cupid's bow were performed, the postoperative pull up of Cupid's bow is probably owed to the scar contracture, which improves by 2 times during the first year after surgery.

## Figures and Tables

**Table 1:** Distribution of complete cleft lip patients according to demographic and anthropometric parameters (*N* = 26).

Patient	Gender	Age (m)	Index of asymmetry		
			T1 (%)	T2 (%)	T1 − T2 (%)
1	Male	32	28	23	5
2	Male	29	30	20	10
3	Female	42	29	18	11
4	Male	23	23	16	7
5	Male	35	43	14	29
6	Male	90	33	12	21
7	Male	46	16	11	5
8	Male	41	32	10	22
9	Female	23	23	9	14
10	Female	21	27	9	18
11	Male	47	12	8	4
12	Male	16	21	7	14
13	Male	40	47	7	40
14	Female	42	23	7	16
15	Male	42	22	6	16
16	Male	21	29	5	24
17	Female	47	26	5	21
18	Female	22	17	5	12
19	Male	40	15	4	11
20	Female	24	15	4	11
21	Female	20	36	4	32
22	Female	34	17	3	14
23	Female	16	11	3	8
24	Female	19	30	2	28
25	Female	32	12	1	11
26	Male	22	22	0.4	21.6

M ± SD		33.3 ± 15.4	24.5 ± 9.2*	8.1 ± 6*	16.2 ± 9.1

M: mean, SD: standard deviation, m: months, T1: 2 to 6 months after surgery, T2: 12 to 14 months after surgery, −: subtraction, *P < 0.01 for the comparison between the periods (T1 > T2).

**Table 2:** Distribution of noncleft individuals according to demographic and anthropometric parameters (*N* = 50).

Patient	Gender	Age (m)	Index of asymmetry (%)
1	F	29	8
2	M	18	7
3	M	21	7
4	M	17	7
5	M	40	7
6	M	43	7
7	F	50	7
8	F	48	7
9	F	46	7
10	M	39	7
11	F	23	6
12	F	39	6
13	F	51	6
14	F	42	6
15	M	42	6
16	M	22	6
17	F	31	5
18	M	11	5
19	F	35	5
20	M	45	5
21	M	54	5
22	M	48	5
23	M	54	5
24	F	47	5
25	M	51	5
26	M	10	4
27	M	35	4
28	F	7	4
29	F	49	4
30	F	40	4
31	F	55	4
32	F	48	4
33	F	40	4
34	M	37	4
35	F	39	4
36	M	23	3
37	M	15	3
38	F	28	3
39	M	48	3
40	M	54	3
41	M	49	3
42	F	45	3
43	F	37	3
44	F	34	2
45	M	54	2
46	M	46	2
47	M	38	2
48	M	54	1
49	F	37	1
50	F	52	0

M ± SD		38.4 ± 13.07	4.52 ± 1.89

M: mean, SD: standard deviation, m: months.
